# Adherence to 2015 ESC Guidelines for the Treatment of Infective Endocarditis: A Retrospective Multicentre Study (LEIOT Study)

**DOI:** 10.3390/antibiotics12040705

**Published:** 2023-04-04

**Authors:** Carlo Pallotto, Cesare Bolla, Serena Penpa, Giovanni Genga, Cristina Sarda, Elisabetta Svizzeretto, Andrea Tommasi, Elisa Stolaj, Andrea Salvaderi, Giorgia Piceni, Antonio Maconi, Guido Chichino, Daniela Francisci, on behalf of the LEIOT Study Group

**Affiliations:** 1 Infectious Diseases Clinic, Santa Maria della Misericordia Hospital, University of Perugia, 06100 Perugia, Italy; 2 Infectious Diseases, Department of Internal Medicine, Azienda Ospedaliera SS, Antonio e Biagio e Cesare Arrigo, 15121 Alessandria, Italy; 3 Research Training Innovation Infrastructure, Research and Innovation Department, Azienda Ospedaliera SS, Antonio e Biagio e Cesare Arrigo, 15121 Alessandria, Italy; 4 Infectious Diseases Clinic, Fondazione IRCCS Policlinico San Matteo, 27100 Pavia, Italy

**Keywords:** infective endocarditis, guidelines, adherence, antibiotic treatment, daptomycin, rifampin, gentamicin

## Abstract

Background: Infective endocarditis (IE) is still a severe disease with elevated morbidity and mortality. Nevertheless, the last European guidelines (GL) date back to 2015, and a recent survey described a diffuse suboptimal adherence to their recommendations. Here, we described a real-life scenario about adherence to IE treatment GL. Methods: This was a retrospective, multicentric, case–control study. All the cases of IE admitted to our wards from 2016 to 2020 were enrolled. Patients were divided into two groups, according to the non-adherence (group A, cases) or adherence (group B, controls) to 2015 ESC guidelines. Only targeted treatments were considered. Groups were compared for demographic, clinical, microbiological, and laboratory data and outcome. As a post hoc analysis, we analysed the characteristics of deviations from the guidelines and how these deviations affected mortality. Results: A total of 246 patients were enrolled, with 128 (52%) in group A and 118 (48%) in group B. Groups were homogeneous except for aetiologies: staphylococcal and blood-culture-negative IE were more frequent in group A, while streptococcal and enterococcal IE were more frequent in group B (*p* < 0.001). In-hospital mortality was comparable in the two groups. The most frequent causes of deviations from the guidelines were use of daptomycin, in addition to standard treatments and the missing administration of rifampin or gentamycin. Conclusions: Adherence to 2015 ESC guidelines was limited but it did not affect mortality.

## 1. Introduction

Infective endocarditis (IE) is still a disease with relatively low incidence but elevated morbidity and mortality [1,2]. Despite the increasing interest about this disease, the last European guidelines date back to 2015 [1]. In the years following, the literature has been enriched by several articles about new antibiotics, such as: fifth-generation cephalosporins and long-acting molecules; combination therapies (i.e., daptomycin plus beta-lactams); and new treatment strategies (oral vs. intravenous therapy) that are now more and more frequently used [3,4,5,6,7,8,9]. Moreover, the different available guidelines sometimes appear not to be consistent with one another, especially for antibiotic treatments [10]. Probably due to these reasons and the lack of solid evidence, international guidelines are not completely followed even by their own authors, as described by Tissot-Dupont and colleagues [11]. The survey underlined how the adherence was low in clinically severe situations such as staphylococcal IE, while it was higher when the protocol was clearer (i.e., streptococcal IE). Unfortunately, in recent years, staphylococci became the most frequently isolated aetiologic agent of IE, both in local and in international studies [12,13,14].

Lack of adherence to national and international guidelines appears to be not limited to the treatment of infective endocarditis. An interesting 2016 Dutch review described this phenomenon across several medical fields and tried to categorise non-adherence in different patterns, such as patient or physician decision, demographics, and contraindications. Interestingly, most deviations did not impact the quality of care or the outcome [15].

A recent Italian survey about the use of a combination of beta-lactams and daptomycin for the treatment of infective endocarditis stated that about one-third of the participating clinicians chose daptomycin as first-line treatment of IE; this percentage rose to 44% when methicillin-resistant *Staphylococcus aureus* (MRSA) was involved [6]. Quite a large amount of literature is now available about daptomycin use in patients with IE due to *S. aureus*, both in monotherapy and in combination with beta-lactams [16,17,18]. The combination with fosfomycin was recently studied with encouraging results in MRSA IE [19] and, in an animal model, also in IE due to methicillin-susceptible *S. aureus* (MSSA) [20]. In addition to this, similar combinations were also studied for streptococcal IE to avoid aminoglycoside administration and the related renal toxicity [5]; the availability of long-acting antibiotics such as dalbavancin opened new possibilities of treatment, especially in an outpatient setting [7]. Until now, these kinds of findings have not been considered in the current ESC guidelines that date back to 2015. As a consequence, a gap between available guidelines and clinical practice has emerged.

To the best of our knowledge, this gap was only described in the literature on a survey level. The aim of this study was to describe the adherence to IE treatment guidelines in a real-world clinical setting and, as a secondary objective, to evaluate the impact of adherence on mortality. The findings of the present study could be helpful for stimulating a revision of the current guidelines. 

## 2. Materials and Methods

This was a retrospective, observational, multicentric, case–control study. We retrospectively enrolled all adult patients with infective endocarditis consecutively admitted to the Infectious Diseases Clinic, the Santa Maria della Misericordia Hospital of Perugia, Italy, and to the Azienda Ospedaliera “SS. Antonio e Biagio e Cesare Arrigo” of Alessandria, Italy, from 2016 to 2020. Exclusion criteria were as follows: (i) age < 18 years; (ii) denial of consent; (iii) length of hospital stay less than 48 h. Demographic, clinical, microbiological, and laboratory data were collected using an electronic ad hoc case report form. Comorbidities were evaluated using the Charlson comorbidity index.

The diagnosis of IE was made in agreement with the 2015 guidelines of the European Society of Cardiology (ESC) [1]. These guidelines were used as reference guidelines to establish adherence. 

The study population was divided into 2 groups: patients treated inconsistently with the guidelines (group A, cases), and patients treated consistently with the guidelines (group B, controls). The two groups were compared to evaluate their characteristics and, as a secondary outcome, their patterns of non-adherence to the guidelines. We defined the “in excess” and “in default” deviations as the addition to or the lack of a drug from the treatment in comparison with ESC guidelines, respectively; a third kind of deviation was indicated when the antibiotic treatment scheme was entirely different from guidelines and was also used for mixed in excess/in default deviations. In addition to this, as a post hoc analysis, we evaluated the impact of adherence to the guidelines on mortality. All the analyses only took into consideration the first targeted therapy administered for at least 3 days. 

The statistical analysis was executed with IBM SPSS Statistics version 23. Continuous non-Gaussian variables were represented as median and interquartile range, while categorical variables were represented as frequency and percentage. Comparison between groups was performed with a two-tailed Mann–Whitney test or a chi-squared test with Yates’ correction, depending on variable distribution. A value of p less than 0.05 was considered statistically significant. According to the retrospective design of the study and to its primary descriptive aim, we did not calculate a sample size. 

All the patients provided a signed consent form for retrospective studies according to the local Ethics Committee recommendations. This study was conducted in line with the principles of good clinical practice and the Declaration of Helsinki. Local Ethics Committees have approved this study.

## 3. Results

We consecutively enrolled 246 patients. In total, 150 (61%) were males, the median age was 73 years (interquartile range (IQR) 61-80 years), and in 118/246 (48%) patients, targeted antibiotic treatment was consistent with 2015 ESC guidelines, while in 128 (52%) patients, a different treatment was administered. Table 1 describes the demographic, clinical, and microbiological characteristics and mortality of the study population, group A (non-adherence to guidelines) and group B (adherence to guidelines). Left-sided IEs (176/246, 72%) were more frequent than other localizations, with no differences between aortic and mitral valve (88/246, 35.8% and 89/246, 36.2% respectively). The majority of patients at admission suffered from a fever (182/246, 74%), while 90/246 (36.6%) patients sought medical attention because of an embolic event. These complications were quite common during the whole hospital stay; at least one embolic event was reported in 126/246 (51.2%) patients, especially affecting the central nervous system (40/246, 16.3%) and vertebral column (36/246, 14.6%). Valvular complications were reported as follows: perforation, abscess, and prosthesis’ dehiscence in 19/246 (7.7%), 23/246 (9.3%), and 9 (3.7%) patients, respectively. Renal insufficiency and heart failure were more frequent (47/246, 19.1% and 49/246, 19.9%, respectively). Staphylococcal IE represented about 41% of cases (102/246). In-hospital mortality was 15.6% (39/246 patients). Group A and group B were homogeneous for age, sex, comorbidities, type of valve involved in the infection, complications (both embolic events and local and systemic complications), surgical treatment, and mortality. In particular, comorbidities such as chronic kidney insufficiency, haemodialysis, predisposition to cardiovascular diseases, and diabetes mellitus did not differ between the two groups either, if evaluated globally by the Charlson comorbidity index. On the other hand, prosthetic valve IEs (PVEs) were significantly more frequent in group A. Staphylococcal and blood-culture-negative IEs were significantly associated to non-adherence to guidelines, while streptococcal and enterococcal IEs were more frequently described in group B. Finally, hospital stay was significantly longer in group A (42 vs. 34 days, *p* = 0.011).

In excess deviations were observed in 86/128 (67.2%) patients; in particular, in 38/86 (44.2%) and 52/86 (60.5%), the “excess” consisted of daptomycin administration when not strictly indicated by guidelines or the addition of an adjunctive antibiotic to the indicated treatment scheme, respectively. In 27/128 (21.1%) cases, an in default deviation was reported; the lack of gentamycin (5/27, 18.5%) or rifampicin (9/27, 33.3%) in the prosthetic valve IE treatment scheme were the main causes of these deviations. The remaining 15/128 (11.7%) patients received a treatment substantially different from the guidelines or a scheme with a mixed in excess/in default deviation.

**Figure 1:**
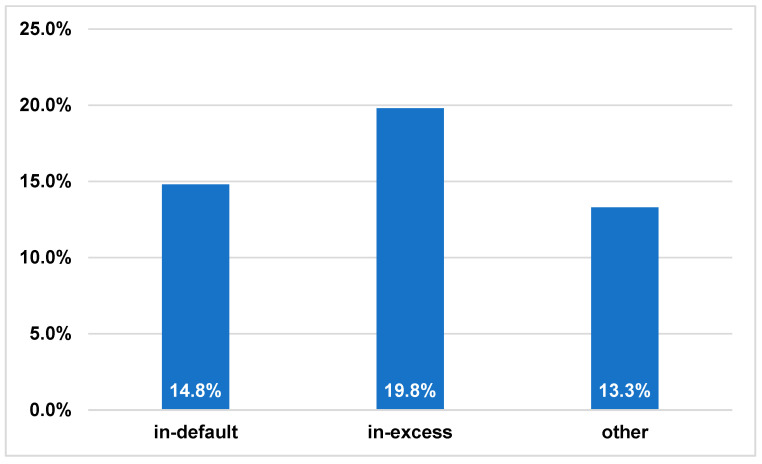
In-hospital mortality distribution for non-adherence type (*p* > 0.1).

As a post hoc analysis, we evaluated the risk factors for in-hospital mortality (Table 2). IE with a pacemaker or intracardiac defibrillator (PMK/ICD) showed a significantly higher mortality rate (*p* = 0.046). Other significant risk factors were complications such as lung embolisation, renal insufficiency and heart failure, or a higher c-reactive protein level at admission (*p* = 0.021, *p* < 0.001, *p* < 0.001, *p* = 0.006, respectively). Both the procalcitonin level at admission and its highest value during the hospital stay (peak) were comparable between the two groups. In-hospital mortality was not affected by treatment adherence to guidelines, *S. aureus* aetiology, or methicillin-resistant *S. aureus* aetiology. Moreover, the in-hospital mortality rate between the different non-adherence types was comparable (14.8%, 19.8% and 13.3% for in default, in excess, and other types of guideline deviations, *p* > 0.1%) (Figure 1).

## 4. Discussion

The 2015 ESC guidelines [1] precisely and extensively described the recommendations for the antibiotic treatment of IE. Nevertheless, a relevant percentage of physicians do not follow these recommendations, as shown by Tissot-Dupont and colleagues in a recent survey [11]. This finding is not surprising. Before the 2015 ESC guidelines were issued, a comparable international survey was conducted by Beraud and colleagues on more than 800 physicians: heterogeneity in the management of IE was very high, especially regarding antimicrobial treatment [21]. More recently, another survey with reference to both European and American guidelines showed similar results [22]. This study evaluated how the results of these surveys reflected physicians’ behaviour in a real-life clinical setting. More than 50% of patients received an antibiotic treatment that differed from the guidelines. Consistently with the abovementioned surveys, the adherence was significantly lower in staphylococcal and blood-culture-negative IE, while in streptococcal cases, it was quite complete. In severe cases, such as staphylococcal IE or PVEs, a personalised treatment could be administered more frequently because clinical presentation and patients’ needs could be more difficult to standardise. For blood-culture-negative IE, local epidemiology and a patient’s risk factors should be substantially taken into consideration. Moreover, the level of evidence that supports guidelines was only elevated for a small percentage of recommendations [22,23]. Another item of interest to understand the scarce adherence to IE guidelines was well analysed in a recent Japanese study. The authors highlighted a certain “gap” between patients eligible for randomised clinical trials on EI and patients not eligible. The latter group consisted of older patients with more comorbidities and a more severe IE [24]. These findings could contribute to at least partially explaining deviations from the guidelines.

Despite significant differences in aetiologies between the two groups, in our study, in-hospital mortality was not affected by adherence to guidelines. Our study was not designed to analyse this fundamental aspect. On the other hand, we think that information about mortality should be provided in this context to have a wider and more concrete view of IE. Moreover, it could be worth highlighting that the overall mortality shown in this study is comparable to that reported in the recent literature [25]. A previous real-life study conducted in Spain with reference to 2004 and 2009 ESC guidelines reported comparable results but with a homogeneous distribution of streptococcal and staphylococcal IE [26].

We also evaluated the characteristics of the deviations from the guidelines. Most in default deviations consisted of a lack of rifampin or gentamicin administration in PVE. The last European guidelines suggested the addition of gentamicin and rifampin to the treatment backbone (cloxacillin for MSSA and vancomycin or daptomycin for MRSA) for staphylococcal PVE cases to increase the bactericidal activity and to reach the dormant bacteria in the biofilm [1]. While rifampin administration is quite heterogeneous—91% of interviewed physicians described using rifampin for PVE in a recent survey from the Unites States [22]—the absence of rifampin is one of the most frequent deviations from guidelines in the abovementioned study published by Tissot-Dupont [11], and aminoglycosides seem to meet less favour. In effect, Lebeaux and colleagues hypothesized that these may not be necessary in staphylococcal PVE [27], and aminoglycoside-sparing regimens were also described for streptococcal IE [5] treatment. Consistent with the literature [11,21,22], our experience found that the main reason for the lack of rifampin or aminoglycoside rested on the risk of adverse events. On the other hand, daptomycin was the primary cause of in excess deviations. This finding is all but surprising. Several papers described daptomycin use in IE, as a single agent or as part of combination therapies [3,5,28,29,30], and different surveys also testified to the wide usage of this drug for IE treatment [6,11,22].

Risk factors for in-hospital mortality were also described as a post hoc analysis. As shown in Table 2, the types of valves involved in IE were significantly associated with mortality, but this finding rested quite entirely on the “other” category. This category consisted of PMK/ICD IEs and pulmonary valves. This association was also confirmed by the significantly higher mortality reported on PMK/ICD IE and patients with an embolic complication of the lung. Consistent with data from the literature [31], complications such as heart failure and renal insufficiency were associated with higher mortality rates. On the other hand, in our study, age and staphylococcal aetiology (neither *S. aureus* nor methicillin-resistant *S. aureus*) were described as related to a poor outcome. These last findings were unexpected and are probably due to the relatively small sample size of the present study.

Finally, adherence to GL was similar in the “alive” and “poor outcome” groups (49.3% vs. 41%, respectively; *p* > 0.1), and mortality was not influenced by the different types of deviation from the GL.

This study presented several limitations, such as its retrospective design and the relatively limited sample size. In addition to this, as previously mentioned, this study was not designed to and does not have enough power to statistically analyse mortality. However, it described a real-life situation, and it could contribute to future evaluation for guideline revisions and updates. 

In conclusion, adherence to IE guidelines for antibiotic treatment was limited but it seemed not to affect patients’ outcomes. Uses of daptomycin, gentamicin, and rifampin were the most frequent causes of deviation from the guidelines. More studies would be necessary to better understand the reasons for deviations. Finally, according to the quite large amount of data from the literature, a more frequent update of international guidelines would be helpful.

## Figures and Tables

**Table 1:** Demographic, clinical, and microbiological characteristics and outcome.

	Total (n = 246)	Non-Adherence (n = 128)	Adherence (n = 118)	*p*
Males—n (%)	150 (61)	76 (59.4)	74 (62.7)	>0.1
Age—year, median (IQR)	73 (61–80)	73 (62–80)	73 (58.75–80)	>0.1
Valve involved—n (%)				>0.1
- Aortic	88 (35.8)	48 (37.5)	40 (33.9)	
- Mitral	89 (36.2)	48 (37.5)	41 (34.7)	
- Tricuspid	22 (8.9)	12 (9.4)	10 (8.5)	
- Plurivalvular	40 (16.3)	17 (13.3)	23 (19.5)	
- other	7 (2.8)	3 (2.3)	4 (3.4)	
Prosthetic valves—n (%)	71 (28.9)	48 (37.5)	23 (19.5)	0.002
PMK/ICD—n (%)	30 (12.2)	17 (13.3)	13 (11)	>0.1
Risk factors and comorbidities—n (%)				
- Previous IE	24 (9.8)	13 (10.2)	11 (9.3)	>0.1
- Predisposing heart condition	139 (56.5)	76 (61.7)	63 (53.4)	>0.1
- Previous cardiosurgical intervention	90 (36.6)	54 (42.2)	36 (30.5)	0.057
- Chronic kidney insufficiency	60 (24.4)	29 (22.7)	31 (26.3)	>0.1
- Haemodialysis	12 (4.9)	7 (5.5)	5 (4.2)	>0.1
- Intravenous drug users	20 (8.1)	10 (7.8)	10 (8.5)	>0.1
- Diabetes mellitus	58 (23.6)	28 (21.9)	30 (25.4)	>0.1
Charlson Comorbidity Index—median (IQR)	5 (3.25–7)	5 (3.75–7)	6 (3.25–7)	>0.1
Characteristics at admission—n (%)				
- Fever	182 (74)	96 (75)	86 (72.9)	>0.1
- TIA/stroke	30 (12.2)	21 (16.4)	9 (7.6)	0.036
- Other embolisations	60 (24.4)	32 (25)	28 (23.7)	>0.1
Aetiology—n (%)				<0.001
- *Staphylococcus* spp.	102 (41.5)	71 (55.5)	31 (26.3)	
- *Streptococcus* spp.	53 (21.5)	18 (14.1)	35 (29.7)	
- *Enterococcus* spp.	30 (12.2)	9 (7)	21 (17.8)	
- Other	26 (10.6)	7 (5.5)	19 (16.1)	
- Blood culture negative	35 (14.2)	23 (18)	12 (10.2)	
Vegetation size >10 mm—n (%)	84/235 (35.7)	46/124 (37.1)	38/111 (34.2)	>0.1
Embolic complications—n (%)	126 (51.2)	65 (50.8)	61 (51.7)	>0.1
- CNS	40 (16.3)	25 (19.5)	15 (12.7)	>0.1
- Spleen	22 (8.9)	12 (9.4)	10 (8.5)	>0.1
- Skin and skin structures	22 (8.9)	13 (10.2)	9 (7.6)	>0.1
- Spondylodiscitis	36 (14.6)	18 (14.1)	18 (15.3)	>0.1
- Liver	7 (2.8)	3 (2.3)	4 (3.4)	>0.1
- Kidney	10 (4.1)	7 (5.5)	3 (2.6)	>0.1
- Lung	39 (15.9)	20 (15.6)	19 (16.1)	>0.1
- Other	35 (14.2)	19 (14.8)	16 (13.6)	>0.1
Other complications—n (%)				
- Valvular perforation	19 (7.7)	12 (9.4)	7 (5.9)	>0.1
- Perivalvular abscess	23 (9.3)	14 (10.9)	9 (7.6)	>0.1
- Dehiscence of the prosthesis	9 (3.7)	7 (5.5)	2 (1.7)	>0.1
- Renal insufficiency	47 (19.1)	27 (21.1)	20 (16.9)	>0.1
- Heart failure	49 (19.9)	28 (21.9)	21 (17.8)	>0.1
Surgical treatment—n (%)	69 (28)	37 (28.9)	32 (27.1)	>0.1
In-hospital mortality—n (%)	39 (15.6)	23 (18)	16 (13.6)	>0.1
Length of hospital stay—days, median (IQR)	38 (24–56.75)	42 (28.75–58.25)	34 (21–52)	0.011

**Table 2:** Risk factors for in-hospital mortality.

	Total (n = 246)	Alive (n = 207)	Poor Outcome (n = 39)	*p*
Males—n (%)	150 (61)	129 (62.3)	21 (53.8)	>0.1
Age—year, median (IQR)	73 (61–80)	73 (60.5–80)	71 (62.5–80)	>0.1
Valve involved—n (%)				0.034
- Aortic	88 (35.8)	77 (37.2)	11 (28.2)	(>0.1)
- Mitral	89 (36.2)	76 (36.7)	13 (33.3)	(>0.1)
- Tricuspid	22 (8.9)	19 (9.2)	3 (7.7)	(>0.1)
- Plurivalvular	40 (16.3)	32 (15.5)	8 (20.5)	(>0.1)
- Other	7 (2.8)	3 (1.4)	4 (10.2)	(0.012)
Prosthetic valves—n (%)	71 (28.9)	58 (28)	13 (33.3)	>0.1
PMK/ICD—n (%)	30 (12.2)	21 (10.1)	9 (23.1)	0.046
Risk factors and comorbidities—n (%)				
- Previous IE	24 (9.8)	18 (8.7)	6 (15.4)	>0.1
- Predisposing heart condition	139 (56.5)	118 (57)	21 (53.8)	>0.1
- Previous cardiosurgical intervention	90 (36.6)	72 (34.8)	18 (46.2)	>0.1
- Chronic kidney insufficiency	60 (24.4)	48 (23.2)	12 (30.8)	>0.1
- Haemodialysis	12 (4.9)	9 (4.3)	3 (7.7)	>0.1
- Intravenous drug users	20 (8.1)	15 (7.2)	5 (12.8)	>0.1
- Diabetes mellitus	58 (23.6)	48 (23.2)	10 (25.6)	>0.1
Charlson Comorbidity Index—median (IQR)	5 (3.25–7)	5 (3.5–7)	6 (3.5–7)	>0.1
Characteristics at admission—n (%)				
- Fever	182 (74)	153 (73.9)	27 (69.2)	>0.1
- TIA/stroke	30 (12.2)	23 (11.1)	7 (17.9)	>0.1
- Other embolisations	60 (24.4)	51 (24.6)	9 (23.1)	>0.1
Laboratory test at admission—mean (± SD)				
- WBC (×10^3^/mm^3^)	9790 ± 5184	9765 ± 5069	9925 ± 5847	>0.1
- Hb (g/dL)	10.8 ± 2	10.9 ± 2	10.4 ± 2.2	>0.1
- PCR (mg/dL)	11 ± 8.1	10.4 ± 8.1	14.4 ± 7.6	0.006
- Peak of PCT (ng/mL)	10.6 ± 25.8	8.9 ± 23.7	17.8 ± 32.9	0.086
- PCT at admission (ng/mL)	4.3 ± 14.5	3.9 ± 15	6.5 ± 11.7	>0.1
Aetiology—n (%)				>0.1
- *Staphylococcus* spp.	102 (41.5)	81 (39.1)	21 (53.8)	(0.087)
- *Streptococcus* spp.	53 (21.5)	46 (22.2)	7 (17.9)	(>0.1)
- *Enterococcus* spp.	30 (12.2)	25 (12.1)	5 (12.8)	(>0.1)
- Other	26 (10.6)	23 (11.1)	3 (7.7)	(>0.1)
- Blood culture negative	35 (14.2)	32 (15.5)	3 (7.7)	(>0.1)
Aetiology, *S. aureus*—n (%)	77 (31.3)	61 (29.5)	16 (41)	>0.1
Aetiology, MRSA—n (%)	20 (8.1)	17 (8.2)	3 (7.7)	>0.1
Vegetation size >10 mm—n (%)	84/235 (35.7)	69/198 (34.8)	15/37 (40.5)	>0.1
Embolic complications—n (%)	126 (51.2)	101 (48.8)	25 (64.1)	0.079
- CNS	40 (16.3)	31 (15)	9 (23.1)	>0.1
- Spleen	22 (8.9)	19 (9.2)	3 (7.7)	>0.1
- Skin and skin structures	22 (8.9)	18 (8.7)	4 (10.3)	>0.1
- Spondylodiscitis	36 (14.6)	34 (16.4)	2 (5.1)	>0.1
- Liver	7 (2.8)	5 (2.4)	2 (5.1)	>0.1
- Kidney	10 (4.1)	6 (2.9)	4 (10.3)	0.091
- Lung	39 (15.9)	28 (13.5)	11 (28.2)	0.021
- Other	35 (14.2)	29 (14)	6 (15.4)	>0.1
Other complications—n (%)				
- Valvular perforation	19 (7.7)	17 (8.2)	2 (5.1)	>0.1
- Perivalvular abscess	23 (9.3)	18 (8.7)	5 (12.8)	>0.1
- Dehiscence of the prosthesis	9 (3.7)	7 (3.4)	2 (5.1)	>0.1
- Renal insufficiency	47 (19.1)	31 (15)	16 (41)	<0.001
- Heart failure	49 (19.9)	26 (12.6)	23 (59)	<0.001
Surgical treatment—n (%)	69 (28)	58 (28)	11 (28)	>0.1
Adherence to therapy guidelines—n (%)	118 (48)	102 (49.3)	16 (41)	>0.1
Length of hospital stay—days, median (IQR)	38 (24–56.75)	40 (25.5–57.5)	27 (17.5–47.5)	0.012

## Data Availability

The datasets analysed during the current study are not publicly available due to privacy protection reasons but are available from the corresponding author on reasonable request.
